# Editorial: Particle manipulation in microfluidic devices

**DOI:** 10.3389/fbioe.2022.1087299

**Published:** 2022-11-30

**Authors:** P. Paiè, A. Volpe, M. Li

**Affiliations:** ^1^ Department of Physics, Politecnico di Milano, Milano, Italy; ^2^ Physics Department, Politecnico di Bari, Bari, Italy; ^3^ Institute for Photonics and Nanotechnologies (IFN), National Research Council, Bari, Italy; ^4^ School of Engineering, Macquarie University, Sydney, NSW, Australia

**Keywords:** sample heterogeneity, microfluidics, lab on a chip, fluidics, microcomponent, imaging, sorting

Biological samples, and more in detail cell populations, are intrinsically heterogeneous, nevertheless standard approaches analyze the average properties of the entire cell populations, hindering single-cell specificity.

Therefore, the development of alternative approaches single-cell investigation is a priority for human health, with several implications in diagnosis, screening as well as in patient monitoring and personalized drug optimization.

This Research Topic composed of five contributions that identify, measure, and analyze sample heterogeneity to optimize system performances.

An example is the work presented by Woo et al., who report the comparison between different protocols for tissue clearing, by Punching Assisted Clarity Analysis (PACA). Using this method, they have been able to compare the efficiency of more than 28 tissue clearing protocols in rodent brain samples. Given the sample heterogeneity, including differences in cell density and in neural and blood vessel networks, they have retrieved clear regional differences in tissue transparency that remained consistent across all tested protocols, irrespective of tissue thickness.

Among different procedures to investigate sample heterogeneity, microfluidics is becoming a powerful instrumentation to target this goal (Yin and Marshall, 2012). Lab-on-a-chip technologies based on microfluidic networks are indeed major allies in single-cell analysis procedures (Haeberle and Roland, 2007). However, this requires the capability to assess particle manipulation, to sort, orient, align and stretch specimens in a controlled way. This comes together with the necessity of performing precise fluid control in terms of pressure, temperature, and fluidic resistance. The remaining four papers of this Research Topic cover these themes.

Talebjedi et al. present the optimization of an acousto-fluidic system for bioparticle separation. Using neural networks with optimization algorithms, they provide a robust optimization platform for microfluidic systems. The proposed methodology has been demonstrated to significantly improve the system performances.

Wenger et al. use microfluidic systems to investigate the lysozyme diffusion in agarose hydrogels. Hydrogels are biocompatible polymer-based materials with a high-water content and their diffusion coefficient is an important property in relation to their final applications. The microfluidic system presented in this work successfully resolved significant differences between several concentrations and types of agarose, while offering low consumption of analytes and hydrogels as well as simple instrumentation.

Telles-Silva et al. reviews how organoids and microfluidic-based organ-on-a-chip technologies can be used to model human liver, as an alternative to animal experimentation and predominant 2D models. Given the tissue complexity of this organ, and the limited efficacy of major 2D *in vitro* models, they discuss how these liver-on-a-chip technologies allow an efficient cellular microenvironment control, resembling *in vivo* cellular responses to drugs.

Strauß et al., have used a fluidic sensor to improve the reproducibility of bioprinting process. In this case, the sample which consists in a heterogeneous hydrogel cell mixture is used as a bioink in an advanced additive manufacturing process to build complex tissue constructs. Using this temperature-based fluidic sensor, they have achieved promising results, such as improved reproducibility and robustness.
